# Histone preconditioning protects against obstructive jaundice-induced liver injury in rats

**DOI:** 10.3892/etm.2014.1697

**Published:** 2014-04-25

**Authors:** YOU-XING ZHOU, YONG NI, YI-BING LIU, XIAOHONG LIU

**Affiliations:** 1 Department of Hepatobiliary Surgery, The First Affiliated Hospital of Shenzhen University, Shenzhen, Guangdong 518035, P.R. China; 2 Department of Hepatobiliary Surgery, Longgang District Central Hospital of Shenzhen, Shenzhen, Guangdong 518116, P.R. China; 3 Department of Geriatric Psychiatry, Wuxi Mental Health Center, Wuxi, Jiangsu 214151, P.R. China

**Keywords:** histone, precondition, obstructive jaundice, liver injury

## Abstract

A major consequence of obstructive jaundice (OJ) in clinical practice is the development of severe liver injury, and at present, no effective treatments have been developed to protect against it. Preconditioning with damage-associated molecular pattern (DAMP) molecules has been demonstrated to protect multiple organs from injury, and histones have been recently identified as DAMP molecules. The aim of the present study was to investigate the protective effect of histone preconditioning against OJ-induced liver injury in rats and the involvement of Toll-like receptors. Rats were administered histone proteins (200 μg/kg; 1 ml) or physiological saline (1 ml) intraperitoneally 24 h prior to being subjected to bile duct ligation (BDL). The serum levels of liver enzymes and bilirubin, as well as the histopathology were analyzed. The mRNA expression of interleukin-6 (IL-6) in the liver tissue was analyzed using quantitative polymerase chain reaction. BDL in the control group caused severe OJ-induced liver injury, as indicated by the significantly elevated levels of liver enzymes and mRNA levels of IL-6, and confirmed by histopathological alterations. However, histone preconditioning significantly ameliorated the OJ-induced liver injury caused by BDL, as shown by an improvement in the levels of liver enzymes, a suppression of IL-6 production, as well as histopathological alterations. Therefore, these results suggested that histone preconditioning is able to protect against OJ-induced liver injury in rats.

## Introduction

Obstructive jaundice (OJ) is a common pathophysiological process that occurs in numerous clinical conditions, including gallstones, stricture of the bile duct and pancreatic cancer. Surgical or interventional decompression is the main treatment strategy for OJ patients (1). In the treatment of OJ patients, severe complications may occur in surgical or interventional decompression. The procedure of decompression itself is not enough to prevent the complications. One of the major consequences of OJ is the development of severe liver injury (2). The mechanisms responsible for the pathogenesis of OJ-induced liver injury remain largely unknown, although inflammatory cell infiltration, microvascular perfusion failure and Toll-like receptor (TLR) activation are reported to be involved (3,4). At present, there are no effective treatments to protect against OJ-induced liver injury, thus, novel therapeutic strategies are urgently required.

Preconditioning is a process where the body is subjected to mild stress in order to increase its resistance to further stresses. It has been associated with increased resistance and protection against numerous types of tissue injuries, including infected thermal injury, ischemia/reperfusion (I/R) injury and hemorrhagic shock (5–8). Notably, hyperthermia preconditioning has been demonstrated to be an effective method to protect against OJ-induced liver injury in rats (9). The results from this study were in accordance with previous studies, which demonstrated that hyperthermic preconditioning enhances the immune response of rats with OJ (10,11).

Previous studies have demonstrated that numerous damage-associated molecular pattern (DAMP) molecules, including high mobility group box 1 (HMGB1), lipoteichoic acid, lipopolysaccharide (LPS) and heat shock protein, have been successfully developed as preconditioning agents (12–15). Furthermore, the protective effect of HMGB1 preconditioning on hepatic I/R injury was found to involve the downregulation of it’s receptor TLR4 (16). It is noteworthy that pretreatment with LPS, lipoteichoic acid and HMGB1 were all demonstrated to have a protective effect against myocardial I/R injury, suggesting that cross-tolerization may occur between different preconditioning agents (12–14). Histones have been previously identified as alarmins or DAMP molecules, which serve as danger signals in the context of the ‘danger model’ to promote activation of the innate immune system in response to several types of tissue injury, including OJ-induced liver injury (17).

Therefore, in the present study it was hypothesized that preconditioning with histones, which are recently identified DAMP molecules, may protect against OJ-induced liver injury and the downregulation of TLR may be involved in this process.

## Materials and methods

### Reagents

Histones obtained from calf thymus (H9250) were purchased from Sigma-Aldrich (St. Louis, MO, USA). TRIzol reagent was purchased from Invitrogen Life Technologies (Carlsbad, CA, USA). The RevertAid First Strand cDNA Synthesis kit was obtained from Fermantas (Beverly, MA, USA). The SYBR-Green kit was purchased from Bio-Rad (Hercules, CA, USA).

### Animals

Adult male Sprague-Dawley (SD) rats were purchased from the Medical Experimental Animal Center of Guangdong Province (Guangzhou, Guangdong, China). Rats were provided with standard rodent chow and water *ad libitum* under a natural day/night cycle. All the experimental protocols were approved by the Animal Ethics Committee of the First Affiliated Hospital of Shenzhen University (Shenzhen, Guangdong, China).

### Experimental protocol

In total, 18 SD rats were randomly divided into three groups, with each group containing six animals. Animals in group 1 underwent sham surgery (sham group). Animals in group 2 underwent bile duct ligation (BDL) 24 h subsequent to physiological saline pretreatment (control group), whilst animals in group 3 underwent BDL 24 h subsequent to histone pretreatment (HPC group).

Prior to surgery, rats were fasted for 12 h with water *ad libitum*. Each rat was weighed and anesthetized with 10% chloral hydrate (300 mg/kg) intraperitoneally. Following a midline incision, the common bile duct was exposed by careful separation from its surrounding soft tissue and a double-ligature with 5–0 silk suture was performed, and the bile duct was sectioned between the ligatures. A two-layer running suture was then used for abdominal closure with 4–0 dexon and 2–0 nylon. The sham animals underwent the same surgical procedure with the exception of ligation and section of the common bile duct. Animals in the control and HPC groups were intraperitoneally administered 1 ml physiological saline and 200 μg/kg histones from calf thymus, respectively, 24 h prior to BDL. All animals were euthanized 14 days subsequent to BDL with an overdose of chloral hydrate.

### Sample collection

A second laparotomy was performed once the animals were anesthetized, 14 days after BDL. Following collection of the blood samples from the inferior vena cava, the liver was carefully dissected from its attachment and totally excised. The blood samples were stored at 4°C for biochemical analysis of total bilirubin (TB), direct bilirubin (DB) and alanine aminotransferase (ALT) levels in the serum. The left lobe of the liver was excised and flushed with physiological saline and then cut into two sections. One section was immediately frozen in liquid nitrogen and stored at −80°C for the measurement of mRNA levels of TLR-4, TLR-9 and interleukin-6 (IL-6), whilst the other section was fixed in 40 g/l paraformaldehyde for histopathological analysis.

### Histopathological observations

Liver tissues from all the experimental animals were fixed in 40 g/l formaldehyde and embedded in paraffin. For histopathological evaluation, 4-mm slides were stained with hematoxylin and eosin. The sections were scored by an experienced hepatopathologist in a blinded manner. The histological activity index (HAI) scoring system has been previously used to evaluate histopathology in BDL rats (18,19). In the present study, a modified HAI scoring system was used, which included the following lesions: piecemeal necrosis, confluent necrosis, focal (spotty) lytic necrosis, apoptosis, focal inflammation and portal inflammation.

The levels of bile duct proliferation were also scored by an experienced hepatopathologist in a blinded manner on a scale between 0 and 2, with 0 denoting absent or mild; 1 moderate and 2 severe proliferation (20). Neutrophils that accumulated in the liver were counted in a blinded manner in 20 randomly selected fields using a microscope (Olympus BX50-32H01; Olympus, Tokyo, Japan; magnification, ×400). The data are expressed as the number of polymorphonuclear neutrophils per high-power field (PMNs/HPF).

### Blood biochemistry

The results from the histopathological analysis were verified biochemically by measuring the serum levels of ALT, TB and DB in each experimental group using an autoanalyzer (Hitachi 7600–020; Hitachi, Tokyo, Japan).

### Quantitative polymerase chain reaction (qPCR)

Rat liver samples (0.1 g/per sample) stored at −80°C were homogenized in 1 ml TRIzol reagent and the total RNA was isolated in accordance with the manufacturer’s instructions. The synthesis of cDNA was performed using the RevertAid First Strand cDNA Synthesis kit. The house-keeping gene β-actin was used as an internal control to analyze the mRNA expression levels of TLR-4, TLR-9 and IL-6. The sequences of the PCR primers were designed based on cDNA sequences from GenBank (http://www.ncbi.nlm.nih.gov/genbank/), and were as follows: TLR-4, forward 5′-CGCTCTGGCATCATCTTCAT-3′ and reverse 5′-CTCCTCAGGTCAAAGTTGTTGC3′; TLR-9, forward 5′-TGAGCTACAACAGCCAGCCA-3′ and reverse 5′-AATGTCATTGTGTGCCAGGC-3′; IL-6, forward 5′-GTCAACTCCATCTGCCCTTCAG-3′ and reverse 5′-GGTCTGTTGTGGGTGGTATCCT-3′.

qPCR was performed using SYBR-Green PCR master mix according to the manufacturer’s instructions and each sample was analyzed in duplicate. The mRNA levels of each of the genes being investigated were quantified using the ABI 7700 Sequence Detection System (Applied Biosystems, Warrington, UK) using the comparative methods. The quantity of mRNA was calculated using the ΔΔCt method. Ct values for each gene were normalized to the Ct value of β-actin (ΔCt = Ct-_β-actin_ - Ct-_target_). The results are presented as mRNA fold change: 2^−ΔΔCt^ (ΔΔCt = ΔCt_-sham_ - ΔCt_-HPC_, in the HPC group or ΔΔCt = ΔCt_-sham_ - ΔCt_-control_, in the control group).

### Statistical analysis

One-way analysis of variance, with subsequent post-hoc least significant difference tests and Bonferroni tests, was used for comparison between the experimental groups with continuous variables. Differences in the distribution of histopathological scores (modified HAI scores, bile duct proliferation scores and PMNs/HPF) between the groups were assessed using the Mann-Whitney U test. P<0.05 was considered to indicate a statistically significant difference. All analyses were performed using SPSS statistical software version 10.0 (SPSS, Inc., Chicago, IL, USA).

## Results

### Macroscopic observations

Animals that underwent sham surgery (sham group) showed no alterations in the clinical conditions, specifically in normal activity, no irritability, no vertical hair, normal body weight, no yellowed tails, no darkened urine and no pale feces. In the control and HPC groups however, 24 h after surgery the clinical conditions of the animals deteriorated, as shown by decreased activity, irritability, vertical hair, body weight loss, yellowed tails, darkened urine and pale feces. All the animals survived until the end of the experiment. Jaundice was observed in the visceral and parietal peritoneum of all animals with the exception of animals in the sham group. Varying degrees of ascites, enlarged livers and dilated bile ducts above the obstruction point were also observed in all animals with the exception of those in the sham group.

### Microscopic observations

**Figure 1:**
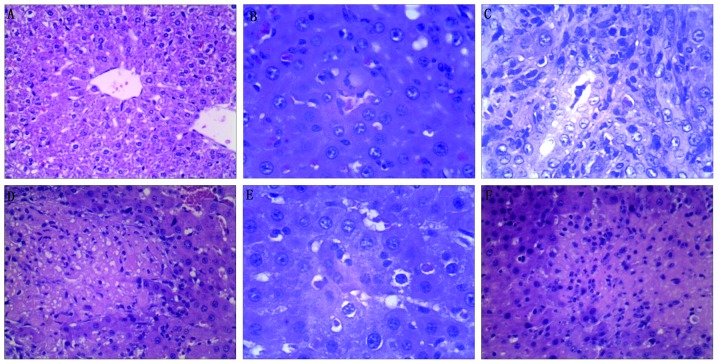
Effects of histone preconditioning on obstructive jaundice-induced liver injury indicated by hematoxylin and eosin staining. (A) In sham rats no histological alterations were observed (sham group). (B) Bile duct ligation in the control group resulted in severe liver injury, demonstrated by an increase in neutrophil infiltration into the liver, as well as an increase in (C) ductal proliferation and (D) severe necroinflammation. Histone preconditioning significantly ameliorated liver injury, shown by the (E) reduction in neutrophil infiltration into the liver and (F) and a decrease in the severity of necroinflammation.

No histological alterations were observed in animals in the sham group. Following euthanasia on day 14 after BDL, severe liver injury was observed in the control group, as indicated by an increase in neutrophil infiltration into the liver tissue, as well as an increase in ductal proliferation and significantly higher modified HAI scores (P<0.05) compared with the sham group. (Fig. 1; Table I).

Histone preconditioning significantly ameliorated the OJ liver injury induced by BDL in the control group, as indicated by a significant reduction in neutrophil infiltration into the liver tissue and decreased modified HAI scores of animals in the HPC group compared with the control group (P<0.05; Fig. 1; Table I). The results of the histopathological analysis, including the PMNs/HPF, the bile duct proliferation scores and the modified HAI scores, of the three groups are summarized in Table 1. No significant difference was identified in the ductal proliferation scores between the HPC group and the control group (P>0.05).

### Blood biochemistry results

**Figure 2:**
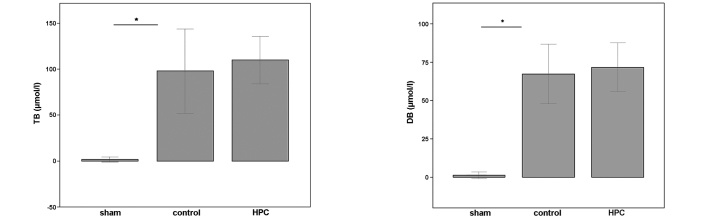
Alterations in serum TB and DB 14 days after BDL and the effect of histone preconditioning. Serum levels of TB and DB in the control group were significantly higher compared with the sham group 14 days subsequent to BDL. Histone preconditioning (HPC group) did not significantly alter the serum levels of TB and DB. ^*^P<0.05, compared with the control using Student’s t-test. TB, total bilirubin; DB, direct bilirubin; BDL, bile duct ligation.

**Figure 3:**
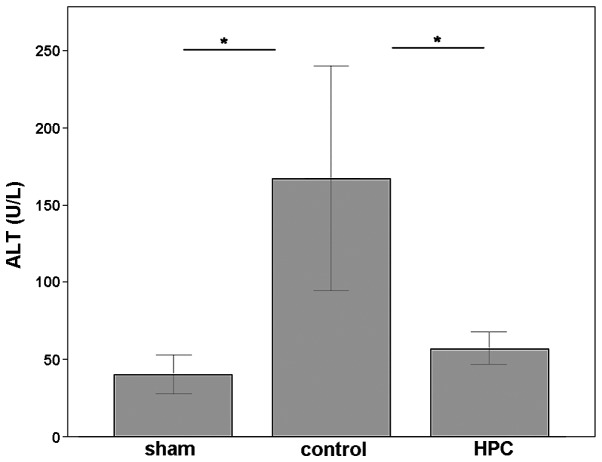
Effect of histone preconditioning on OJ-induced liver injury indicated by blood biochemistry analysis. OJ liver injury induced by BDL was marked in the control group, demonstrated by the significantly increased serum levels of ALT compared with the sham group. Histone preconditioning significantly ameliorated the OJ liver injury induced by BDL. *P<0.05, compared with the control group. OJ, obstructive jaundice; BDL, bile duct ligation; ALT, alanine aminotransferase.

BDL in the control group resulted in significantly elevated serum levels of TB and DB (Fig. 2) compared with the sham group, which suggests that the experimental OJ model was successfully induced. The serum levels of TB and DB in rats preconditioned with histone proteins prior to being subjected to BDL were not significantly different from those in the control animals (P>0.05), indicating that the degree of cholestasis was similar in the two experimental groups (Fig. 2). OJ liver injury induced by BDL was prominent in the control group, as shown by the significantly increased serum levels of ALT (Fig. 3) compared with the sham group (P<0.05). In accordance with the results of histopathological studies, histone preconditioning significantly ameliorated OJ-induced liver injury induced by BDL. The serum levels of ALT were significantly lower than that of the control group (P<0.05; Fig. 3).

### mRNA expression of IL-6, TLR-4 and TLR-9

**Figure 4:**
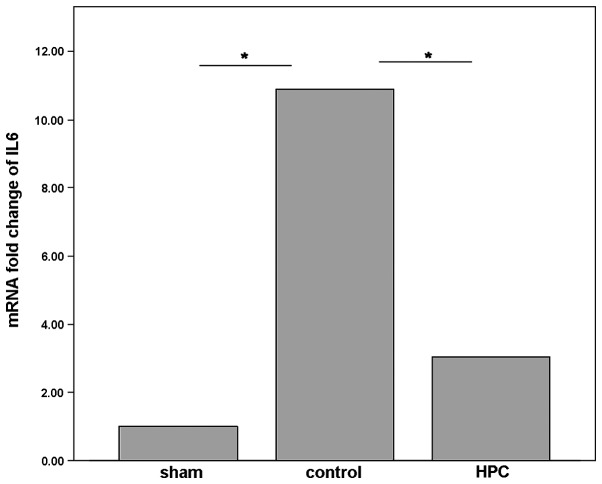
mRNA expression of IL-6 in the liver tissue. BDL in the control group significantly upregulated the mRNA expression of proinflammatory cytokine IL-6 in the liver tissue. Histone preconditioning (HPC group) significantly ameliorated OJ liver injury induced by BDL, as indicated by the decreased mRNA expression levels of IL-6 in the liver tissue. ^*^P<0.05, compared with the control group. IL-6, interleukin-6; BDL, bile duct ligation; OJ, obstructive jaundice.

**Figure 5:**
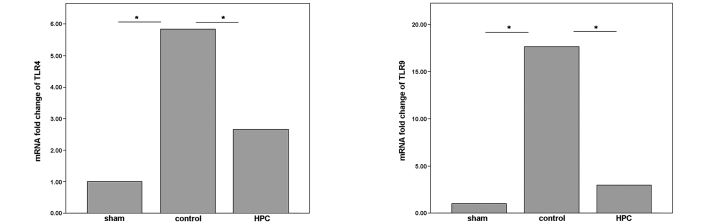
mRNA expression levels of TLR-4 and TLR-9 in the liver tissue. Bile duct ligation in the control group significantly upregulated the mRNA expression levels of TLR-4 and TLR-9 in the liver tissue, while histone preconditioning (HPC group) significantly decreased the mRNA expression levels of TLR-4 and TLR-9 in the liver tissue. ^*^P<0.05, compared with the control group. TLR, Toll-like receptor.

Using qPCR, it was demonstrated that the mRNA expression levels of IL-6 were significantly upregulated by BDL in the control group compared with the sham group (P<0.05; Fig. 4), which is consistent with previous studies (21). However, compared with the control group, histone preconditioning (HPC group) significantly downregulated the mRNA expression levels of IL-6 (P<0.05; Fig. 4). In addition, BDL in the control group significantly upregulated the mRNA expression levels of TLR-4 and TLR-9 (P<0.05; Fig. 5) compared with the sham group, which is consistent with previous studies (4). In the present study it was demonstrated that histone preconditioning significantly ameliorated the upregulation of the mRNA expression levels of TLR-4 and TLR-9. Animals preconditioned with histones expressed significantly lower mRNA levels of TLR-4 and TLR-9 (P<0.05; Fig. 5) compared with animals in the control group.

## Discussion

OJ is a common clinical condition, which has been extensively studied, and is capable of inducing severe liver injury (2). Surgical, endoscopic and interventional decompressions are the primary treatment strategies for patients with OJ. However, biliary intervention has been demonstrated to augment inflammatory cell infiltration and aggravate OJ-induced liver injury (22,23). Preconditioning with DAMP molecules has been demonstrated to be effective in protecting organs from injury in stressed situations (12–15). Preconditioning with hyperthermia (<42°C for 20 min) 12 h prior to being subjected to BDL was found to significantly ameliorate OJ-induced liver injury (9,10). However, hyperthermia is difficult to achieve in the clinic, thus, an effective pharmaceutical drug would be the preferred option.

Histones are a newly identified DAMP molecule, however, the preconditioning effect of histones on OJ-induced liver injury remains to be elucidated. In the present study, the effect of histone preconditioning on OJ-induced liver injury in rats was investigated. Preconditioning with HMGB1 (20 μg/mouse) has been demonstrated to significantly protect against hepatic I/R injury, and preconditioning with LPS (100 μg/kg) has been demonstrated to significantly protect against hepatic I/R injury, so in our study we preconditioned experimental animals with 200 μg/kg histone proteins (16,24). The present study demonstrated that liver injury in animals in the HPC group was significantly ameliorated by histone preconditioning compared with the control group, as indicated by significant differences in the degree of necroinflammation and a decrease in the number of neutrophils infiltrating into the liver tissue. The serum levels of ALT were significantly lower in the HPC group compared with the control group, indicating that necrosis of hepatocytes was prevented, which is consistent with the results from the histopathological analysis.

Inflammation has been revealed to be important in the development of OJ-induced liver injury (2). In addition, the proinflammatory cytokine IL-6 has been demonstrated to be important in the pathogenesis of OJ-induced liver injury (21). Endotoxemia has been found to occur in patients with jaundice and experimental OJ animals, and LPS was demonstrated to be capable of inducing the release of proinflammatory cytokines, including TNF-α and IL-6 (25,26). Therefore, in the present study, the expression levels of IL-6 in each group were investigated. Using qPCR, it was demonstrated that the mRNA expression levels of IL-6 in the control group were significantly higher compared with the sham group, whilst histone preconditioning significantly downregulated the mRNA expression of IL-6. The present study demonstrated that histone preconditioning protects against OJ-induced liver injury in rats by inhibiting the release of inflammatory mediators.

The preconditioning effects of HMGB1 and LPS on hepatic I/R injury were previously reported to be dependent on TLR4 expression (the receptor for HMGB1 and LPS). The receptors for histones have been demonstrated to be TLR-4 and TLR-9, therefore, the present study investigated the role of TLR-4 and TLR-9 in the process of histone preconditioning on OJ-induced liver injury (16,17,24,27). The results from the qPCR analysis revealed that the mRNA expression levels of TLR4 and TLR9 in the liver tissue were significantly lower compared with the control group, which indicated that TLR4 and TLR9 may be involved in the process of histone preconditioning in OJ-induced liver injury.

Histone proteins are assembled with DNA to form nucleosomes in the nucleus and histone modifications have been demonstrated to be important in gene transcription (28,29). Recently, histone proteins have been identified as DAMP molecules (17,27). Furthermore, the present study demonstrated that histone proteins may be used as a preconditioning agent to protect against OJ-induced liver injury. Preconditioning may be achieved with a variety of stress responses and the most widely studied method is ischemia (30). The adaptive responses to preconditioning may enhance the body’s tolerance to further stresses. Although the exact mechanism of preconditioning has not yet been fully elucidated, the activation of potassium channels, metabolic alterations and the generation of nitric oxide have been suggested to be involved (30–33). Investigations into the mechanisms underlying the protective effects of preconditioning have led to the application of DAMP molecules as preconditioning agents to protect against organ injury. However, for inflammatory diseases, for example OJ-induced liver injury, the interactions between different DAMP molecules, the network of proinflammatory cytokines and downstream signaling molecules are complex. Therefore, further studies are required to elucidate the mechanisms and the safety of the preconditioning molecules, particularly as DAMP molecules are known to be toxic.

In conclusion, histone preconditioning protects against OJ-induced liver injury in rats, and TLR4 and TLR9 may be involved in this process. Histone preconditioning may be a novel and promising therapeutic strategy for the treatment of OJ-induced liver injury and possibly other diseases. However, its safety and underlying mechanisms have not been fully elucidated. Thus, further investigations are necessary to ascertain the safety and therapeutic mechanisms underlying the protective effects of histone preconditioning.

## Figures and Tables

**Table I:** Histopathological score of ductal proliferation, modified HAI and the number of PMNs/HPF in the three groups.

	Sham	HPC	Control
Ductal proliferation	0	1	2.0^a^
PMNs/HPF	0	2	7.5^a^,^b^
HAI	1.5	5	9.5^a^,^b^

OJ liver injury induced by BDL was prominent in the control group, shown by the increase in neutrophil infiltration into the liver tissue, as well as an increase in ductal proliferation and significantly higher modified HAI scores. Histone preconditioning significantly ameliorated the OJ liver injury induced by BDL, as indicated by the significant decrease in neutrophil infiltration into the liver tissue and lower modified HAI scores.

a P<0.05, compared with the sham group;

b P<0.05, compared with the HPC group.

OJ, obstructive jaundice; BDL, bile duct ligation; HAI, histological activity index; PMNs/HPF, polymorphonuclear neutrophils per high-power field.
